# The need to scale up HIV indicator condition-guided testing for early case-finding: a case-control study in primary care

**DOI:** 10.1186/s12875-016-0556-2

**Published:** 2016-11-17

**Authors:** Ivo K. Joore, Denise E. Twisk, Ann M. Vanrolleghem, Maria de Ridder, Suzanne E. Geerlings, Jan E. A. M. van Bergen, Ingrid V. van den Broek

**Affiliations:** 1 Department of General Practice, Division Clinical Methods and Public Health, Academic Medical Center, Meibergdreef 9, 1100 DE Amsterdam, The Netherlands; 2 National Institute for Public Health and the Environment (RIVM), Epidemiology & Surveillance Unit, Centre for Infectious Disease Control, Bilthoven, The Netherlands; 3 Department of Medical Informatics, Erasmus University Medical Center, Rotterdam, The Netherlands; 4 Department of Internal Medicine, Division of Infectious Diseases, Academic Medical Center, Amsterdam, The Netherlands; 5 STI AIDS Netherlands (SOA AIDS Nederland), Amsterdam, The Netherlands

**Keywords:** Case-control studies, General practice, HIV infections/prevention & control, HIV infections/epidemiology, Primary healthcare

## Abstract

**Background:**

European guidelines recommend offering an HIV test to individuals who display HIV indicator conditions (ICs). We aimed to investigate the incidence of ICs in primary care reported in medical records prior to HIV diagnosis.

**Methods:**

We did a cross-sectional search in an electronic general practice database using a matched case-control design to identify which predefined ICs registered by Dutch GPs were most associated with an HIV-positive status prior to the time of diagnosis.

**Results:**

We included 224 HIV cases diagnosed from 2009 to 2013, which were matched with 2,193 controls. Almost two thirds (*n* = 136, 60.7%) of cases were diagnosed with one or more ICs in the period up to five years prior to the index date compared to 18.7% (*n* = 411) of controls. Cases were more likely to have an IC than controls: in the one year prior to the index date, the odds ratio (OR) for at least one condition was 11.7 (95% CI: 8.3 to 16.4). No significant differences were seen in the strength of the association between HIV diagnosis and ICs when comparing genders, age groups or urbanisation levels. There is no indication that subgroups require a different testing strategy.

**Conclusions:**

Our study shows that there are opportunities for IC-guided testing in primary care. We recommend that IC-guided testing be more integrated in GPs’ future guidelines and that education strategies be used to facilitate its implementation in daily practice.

**Electronic supplementary material:**

The online version of this article (doi:10.1186/s12875-016-0556-2) contains supplementary material, which is available to authorized users.

## Background

In 2014, UNAIDS set the challenging goal to end the AIDS epidemic by 2030 [1]. UNAIDS stated that worldwide in 2020, 90% of all people living with HIV should be aware of their HIV status, 90% of all diagnosed HIV infected people should receive antiretroviral therapy, and of that group 90% should achieve viral suppression [1].

By the end of 2014, 19,382 persons in the Netherlands, or 88% of the estimated total number of persons living with HIV, had been diagnosed with the infection. In total, 16,821 had started treatment, and of these 15,463 had achieved viral suppression [2]. Our country is thus close to achieving the UNAIDS ’90-90-90’ target of 73% of all people with HIV virally suppressed [1]. However, in recent years there are still approximately 1000 newly diagnosed HIV infections in the Netherlands each year [2]. In 2014, 44% of newly diagnosed persons presented late for care (CD4 count < 350 cells/mm^3^ or with an AIDS-defining event regardless of CD4 count) [2].

The insight of starting treatment as prevention, demonstrated public health benefit of early treatment [3]. Recently, data appeared that proved that initiating antiretroviral therapy after HIV diagnosis regardless of CD4 count improved the health prospects of the person being treated [4, 5]. These findings reinforce the need to detect HIV as early as possible and the importance of linkage to care.

People who are unaware of their infection play an important role in maintaining the HIV epidemic [6]. Dutch general practitioners (GPs) act as gatekeeper to clinical care and are an important point of referral into specialised HIV care [7]. Every Dutch person is required to register with a GP and 75% contacts their GP at least once per year [8]. About two thirds of sexually transmitted infection (STI) care is provided by GPs and one third by STI clinics [7, 9]. The costs of STI tests and medication at the GP are covered by mandatory health insurance, only after the out-of-pocket maximum has been reached [10]. One third of all HIV infections in the Netherlands are diagnosed by GPs [2]. A Dutch study also showed that GPs reach groups of persons at risk of HIV that are not reached by STI clinics, which underlines the important role of primary care in fighting the HIV epidemic [2, 11]. In several European countries, primary care is a setting for creating opportunities to detect HIV as early as possible [11–14].

Dutch GP guidelines already promote provider-initiated HIV testing among high-risk populations, but this strategy alone shows limits in the implementation to detect HIV-infected people [11]. For example, GPs may find it difficult to have complex conversations about sexual risk behaviour [15, 16]. The deployment of the HIV indicator condition (IC)-guided testing strategy in primary care is a new development that was included in an update of Dutch STI guidelines for GPs that appeared at the end of 2013 [17]. ICs are defined as conditions that are associated with a prevalence of undiagnosed HIV in excess of 0.1% and AIDS-defining conditions [18, 19].

A UK case-control study in primary care found multiple ICs that were associated with a subsequent HIV diagnosis [20].

A Dutch case-control pilot study conducted in a higher HIV-prevalence area showed that one or more ICs were observed prior to diagnosis among cases compared to controls [21]. The results of this study cannot be extrapolated to the Dutch national level, as it was performed in six general practices that were located in a higher HIV-prevalence area.

The aim of the present study was to investigate the incidence of ICs reported in medical records up to five years prior to HIV diagnosis among HIV cases compared to matched controls using a primary care database representative of the general population.

## Methods

We carried out a retrospective population-based nested case-control study; data were retrieved from electronic patient medical records (EMRs).

### Data source

The Integrated Primary Care Information (IPCI) database is a longitudinal primary care research database managed at the Erasmus University Medical Center in Rotterdam, the Netherlands [22, 23]. This database contains the EMRs of 747 Dutch GPs [23]. The EMRs contain demographic information (gender, date of birth and living area) and medical information routinely collected during consultations, including laboratory results, medical diagnoses, prescriptions and treatments. The standard information recorded in EMRs does not include sexual orientation or ethnicity. All patient information in the IPCI database is anonymous. The database incorporates a considerable proportion (about 10%) of the total Dutch population, making it representative of the general population, with some underrepresentation of elderly people living in nursing homes [24]. The Supervisory Board of the IPCI database approved the use of IPCI data for our study. Approval by an ethics committee was not necessary for this retrospective study because only anonymous data were used. The researchers had no access to the identity the patients or the GPs.

### Study population

The study population from which cases and controls were extracted consisted of all persons in the database who were 18 years or older and registered at one of the GP practices during the period from 1 January 2009 up to 31 December 2013 (total of five years).

Cases were defined as HIV-positive individuals. If no HIV diagnosis was recorded, an individual could act as control.

Potential cases were selected by extracting the International Classification of Primary Care (ICPC) B90 code ‘HIV infection’ from the EMRs [25]. In addition, EMRs that contain the words ‘HIV’ and ‘AIDS’ in the open text evaluation fields were extracted. EMRs with a B25 code (‘fear of HIV/AIDS’) but without a B90 code were excluded, because these were people who had visited their GP for an HIV test but were not diagnosed with HIV.

Potential cases were manually validated. The extraction of potential cases was performed in duplicate by two of the project’s researchers (IKJ and DT). Discrepancies were discussed until consensus was reached. Uncertain potential cases were presented to a third researcher (IvdB). Cases were matched to controls (up to a maximum of 10) for age, gender and registration at the same general practice in the same calendar year and at least one year of registration time (follow-up time) prior to the index date (date of HIV diagnosis in cases and matched date in the control group). Controls were selected from persons without an ICPC B90 code in their EMRs. Cases could be selected as controls until the year before they became a case. The number of selected controls per case depended on the availability. Selecting as many controls as possible per case ensures maximum statistical power in matched case-control studies to detect differences in the occurrence of rare events [26].

For the confirmed cases within the study period (2009–13) and their matched controls, a history of five years of patient EMRs of validated registration time before the index date was included if available, but at least one year was required (i.e. practices are included in the IPCI database after data collection checks; information or EMRs prior to validation are only included as ‘patient history’, and are not counted as validated registration time). The inclusion of the cases was limited by the start date of the GP participation in IPCI or by the date the patient registered with the GP prior to the index date (date of HIV diagnosis).

### HIV indicator conditions

A total of 26 predefined ICs were selected from the list of ICs with an associated (undiagnosed) HIV prevalence of >0.1% (see Table 1) [18, 21]. The selection criteria for inclusion ICs were described in an earlier pilot study and included only conditions that Dutch GPs may diagnose themselves (as opposed to the ones diagnosed by medical specialists/in secondary care) [21]. Conditions that are unlikely to be diagnosed by a GP were excluded. Where necessary, specific case definitions (‘operational definitions’) were developed for manual searching in the EMRs of the IPCI database. Also, for each IC ICPC code, common medical terminology, abbreviations and synonyms used by the GP or the patient were marked in the EMRs.

**Table 1:** Operational definitions of HIV indicator conditions

HIV indicator conditions [18]	Operational definition of predefined HIV indicator conditions used for search in the EMRs*
Sexually transmitted infections	Chlamydia, gonorrhoea, syphilis, hepatitis B, genital herpes, lymphogranuloma venereum, condyloma acuminata and trichomoniasis
Chlamydia	Chlamydia
Gonorrhoea	Gonorrhoea
Syphilis	Syphilis
Acute or chronic Hepatitis B	Hepatitis B
Genital Herpes	Genital herpes
Lymphogranuloma venereum	Lymphogranuloma venereum
Condyloma acuminata	Condyloma acuminata
Trichomoniasis	Trichomoniasis
Hepatitis A	Hepatitis A
Acute or chronic Hepatitis C	Hepatitis C
Herpes zoster	Herpes zoster
Severe or atypical psoriasis	Psoriasis
Seborrheic dermatitis/exanthema	Seborrhoeic dermatitis
Cervical dysplasia	Cervical dysplasia
Community-acquired pneumonia	Pneumonia
Unexplained oral candidiasis	Oral candidiasis
Mononeuritis	Mononeuritis
Peripheral neuropathy	Peripheral neuropathy
Mononucleosis-like illness	Mononucleosis-like illness is defined as illness with at least two of these symptoms: rash, fever and swollen lymph glands, with or without muscle aches, sore throat and feeling sick.
Unexplained fever	Fever
Unexplained weight loss	Weight loss
Unexplained lymphadenopathy	Lymphadenopathy
Unexplained chronic diarrhoea	Diarrhoea
Unexplained leukocytopenia lasting > 4 weeks	Leukocytopenia
Unexplained thrombocytopenia lasting > 4 weeks	Thrombocytopenia
Unexplained chronic renal impairment	Chronic renal impairment

*EMR Electronic medical record

A distinction was made between the search for specific diagnoses and that for more general/common symptoms. ICs defined as diagnoses were searched for in the complete text of the EMR, while the symptoms were only searched for in the open text evaluation fields of the EMRs, where most of the important symptoms are reported.

A mononucleosis-like illness was considered if at least two of the following symptoms were present in the EMR’s open evaluation text fields: fever, swollen lymph glands and rash with or without pharyngitis, muscle aches and feeling sick. If there were symptoms in the evaluation text fields that might indicate a mononucleosis-like illness, we also searched the other text fields for validating evidence.

The duration of the search period was one to five years prior to the index date, depending on the availability in the EMR. The history time of a control was matched to the history time of its cases, so that cases did not have a history time different from that of their matched controls.

The incidence of newly diagnosed ICs in the five years prior to index date was classified as 1) yes recorded (at least once); 2) absent; and 3) uncertain. Incident ICs and their date of diagnosis were manually transferred into a validation tool. If a person had multiple consultations for the same IC, we used the most recent one before the index date that was recorded by the GP. The search for incidence ICs was carried out in duplicate by the same two researchers (IKJ, DT). They were not blinded for HIV diagnosis in the EMRs as this was not possible due to technical limitations. A third researcher (IvdB) was consulted if consensus could not be reached or if ICs were classified as uncertain.

### HIV testing in persons diagnosed with STIs

According to the Dutch GP guidelines, persons who are diagnosed with chlamydia, gonorrhoea, syphilis or hepatitis B should be advised to have an HIV test. [17] We did a search in the EMRs in the case-control dataset to find out whether patients diagnosed with STIs had been tested for HIV in the same or follow-up consultation(s) within a time window of three months.

### Statistical analyses

We performed conditional logistic regression to investigate whether having at least one STI or IC prior to the index date was associated with testing HIV positive. We performed exact conditional logistic regression to investigate whether specific ICs prior to the index date were associated with testing HIV positive. For very small or zero incidence rates among the cases or controls, odds ratios (ORs) were estimated with ‘median unbiased estimate’. Sensitivity analyses were performed, comparing the associations between ICs and HIV diagnosis when including versus not including unconfirmed (classified as ‘uncertain’) ICs. Furthermore, the interaction of presence of any IC with gender, age group and urbanisation level was tested in a conditional logistic regression model.

Confidence intervals for the percentages of HIV testing in STI diagnosed patients were obtained by Wilson score method [27].

Analyses were performed with SPSS (version 19.0, IBM, USA) except for the exact logistic regression, for which STATA (version 13.1, College Station, TX) was used.

## Results

### Patient characteristics of cases and controls

**Fig. 1:**
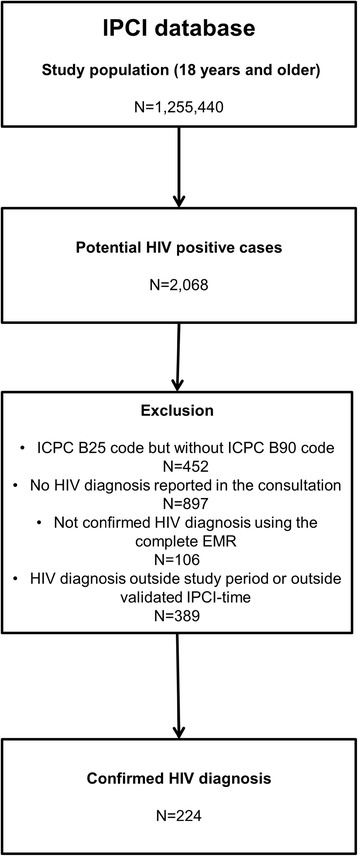
Number of confirmed HIV diagnosis in the IPCI database 2009-2013

The eligible study population (18 years and older) consisted of the EMRs of 1,255,440 persons (Fig. 1). The search algorithm identified 2,068 patients as potential HIV-positive cases. First, patients with a B25 code (‘fear of HIV/AIDS’) but without a B90 code (‘HIV infection’) were excluded (*n* = 452). Of the remaining 1,616 potential cases, 897 patients were excluded because no HIV diagnosis was reported in the consultation. For the 719 potential cases a more in-depth evaluation was conducted using the complete EMR to confirm HIV diagnosis; the diagnosis was confirmed in 613 cases. Patients with an HIV diagnosis outside the study period (*n* = 319) or outside the validated IPCI time (*n* = 70) were excluded.

In total, 224 cases from 135 general practices were matched with 2,193 controls (HIV-negative individuals). The majority (88.4%) of cases were male, the median age was 40 (IQR 31 to 47) and 55.8% lived in areas with a high level of urbanisation (Table 2).

**Table 2:** Patient characteristic of cases and matched controls

	Cases		Controls	
	N	%	N	%
Total study population	224		2,193	
*History prior to index date*				
Between 1 and 2 years	30	13.4	289	13.2
Between 2 and 3 years	27	12.1	270	12.3
Between 3 and 4 years	7	3.1	70	3.2
Between 4 and 5 years	15	6.7	150	6.8
More than 5 years	145	64.7	1,414	64.5
*Gender*				
Male	198	88.4	1,933	88.1
Female	26	11.6	260	11.9
*Age at index date*				
18 to 39 years	80	35.7	788	35.9
40 to 49 years	97	43.3	946	43.1
50 to 59 years	32	14.3	309	14.1
60 years and older	15	6.7	150	6.8
*Urbanisation level*				
very high (>2,500 addresses per km2)	76	33.9	633	28.9
high (1,500 - 2,500 addresses per km2)	49	21.9	522	23.8
medium (1,000 - 1,500 addresses per km2)	21	9.4	252	11.5
low (500 - 1,000 addresses per km2)	9	4.0	113	5.2
not urban/rural (<500 addresses per km2)	4	1.8	61	2.8
Missing	65	29.0	612	27.9
*Socially deprived area (on 4-digit postal code area)* ^a^				
Yes	26	11.6	226	10.3
No	198	88.4	1,967	89.7

^a^Socially deprived areas are defined by various socioeconomic indicators determined per 4-digit postal code

### HIV indicator conditions

Almost two thirds (*n* = 136, 60.7%) of cases had been diagnosed with one or more ICs up to five years prior to the index date, compared to 18.7% (*n* = 411) of controls (Table 3).

**Table 3:** The proportion of persons diagnosed with one or more STI or HIV indicator condition among HIV cases compared to matched controls

	Cases (*N* = 224)		Controls (*N* = 2,193)					
	N	%	N	%	OR	95 % CI		*P* value
*Number of STIs per person in one year prior to index date*								
None	174	78.1	2,169	98.9				
At least one	50	21.9	24	1.1	32.2	17.7 to 58.5		**<0.0001**
*Number of STIs per person up to five years prior to index date*								
None	142	63.4	2,125	96.9				
At least one	82	36.6	68	3.1	25.3	16.1 to 39.9		**<0.0001**
*Number of HIV indicator conditions per person one year prior to index date*								
None	125	55.8	2,032	92.7				
At least one	99	44.2	161	7.3	11.7	8.3 to 16.4		**<0.0001**
*Number of HIV indicator conditions per person up to five years prior to index date*								
None	88	39.3	1,782	81.3				
At least one	136	60.7	441	18.7	8.1	5.9 to 11.1		**<0.0001**

Statistical significance indicated in bold

Cases had a higher probability of having IC than controls: in the one year prior to the index date, the OR for at least one condition compared to no condition was 11.7 (95% CI: 8.3 to 16.4) (Table 3).

Recorded STI episodes in the one year prior to the index date were also associated with the occurrence of HIV: for at least one STI compared to no STI the OR was 32.2 (95% CI: 17.7 to 58.5). The proportions of the number of STIs and ICs per person prior to index date are reported in Additional file 1.

The ICs most strongly associated with the occurrence of HIV were gonorrhoea (OR = 77.1, 95% CI: 27.3 to 300.3) and syphilis (OR = 52.0, 95% CI: 17.6 to 208.2) (Table 4). Several ICs were not observed in either the cases or the control group, i.e. chronic renal impairment, hepatitis A and leukocytopenia.

**Table 4:** The incidence of clinical- and symptoms-related HIV indicator conditions among HIV cases compared to matched controls

	Cases (*N* = 224)		Controls (*N* = 2,193)				
	N	%	N	%	OR	95% CI	*P* value
*Clinical diagnosis*							
Gonorrhoea	33	14.7	5	0.2	77.1	27.3 to 300.3	**<0.0001**
Syphilis	22	9.8	6	0.3	52.0	17.6 to 208.2	**<0.0001**
Leukocytopenia*	2	0.9	0		24.1	1.9 to ∞*	**0.02**
Trichomoniasis	2	0.9	1	0	20.0	1.0 to 1,179.9	0.05
Hepatitis B	9	4.0	4	0.2	22.5	6.3 to 100.0	**<0.0001**
Hepatitis C	3	1.3	2	0.1	15.0	1.7 to 179.6	**0.01**
Thrombocytopenia	4	1.8	4	0.2	12.0	2.0 to 83.1	**<0.01**
Lymphogranuloma venereum	1	0.4	1	0	10.0	0.1 to 785.0	0.35
Mononeuritis	1	0.4	1	0	10.0	0.1 to 785.0	0.35
Cervical dysplasia	6	2.7	11	0.5	8.1	2.0 to 34.6	**<0.01**
Chlamydia	20	8.9	28	1.3	8.5	4.2 to 17.0	**<0.0001**
Genital herpes	8	3.6	7	0.3	10.2	3.1 to 33.7	**0.0001**
Condyloma acuminata	19	8.5	25	1.1	7.9	4.0 to 15.1	**<0.0001**
Oral candidiasis	3	1.3	7	0.3	4.5	0.7 to 21.3	0.11
Mononucleosis-like illness	3	1.3	6	0.3	4.9	0.8 to 23.1	0.09
Pneumonia	25	11.2	79	3.6	3.6	2.1 to 5.9	**<0.0001**
Herpes zoster	10	4.5	32	1.5	3.2	1.4 to 6.9	**<0.01**
Chronic renal impairment*	0	0	3	0.1	2.6	0 to 24.2	1.00
Hepatitis A*	0	0	4	0.2	1.9	0 to 15.1	1.00
Psoriasis	6	2.7	34	1.6	1.8	0.6 to 4.5	0.30
Seborrhoeic dermatitis	7	3.1	60	2.7	1.2	0.4 to 2.8	0.83
*Symptoms*							
Weight loss	7	3.1	19	0.9	3.9	1.3 to 10.1	**0.01**
Lymphadenopathy	9	4.0	31	1.4	3.2	1.3 to 7.3	**0.01**
Fever	8	3.6	29	1.3	2.8	1.1 to 6.3	**0.03**
Diarrhoea	18	8.0	79	3.6	2.5	1.4 to 4.4	**<0.01**
Peripheral neuropathy	5	2.2	25	1.1	0.7	0.1 to 2.8	0.95

OR estimated with ‘Median Unbiased Estimate’ displayed with an asterisk (*). ∞ = infinity

Statistical significance indicated in bold

When uncertain diagnoses were included in the reported ICs, lymphogranuloma venereum (OR = 20.0; 95% CI: 1.0 to 1179.9) was also associated with the occurrence of HIV diagnosis.

The OR for the occurrence of ICs was higher for men than for women, but the difference was not significant (see Table 5). Age group or urbanisation level also had no significant effect on the strength of the association between HIV diagnosis and ICs.

**Table 5:** Association of HIV diagnosis and HIV indicator conditions by gender, age group or urbanisation level

	Cases	Controls			OR^d^	95% CI	*P* value
	n/N (%)^a^	n/N (%)					
							for interaction
Total	136/224	60.7	411/2,193	18.7			
Sex							
Male	122/198	61.6	348/1,933	18.0	8.8	6.3 to 12.3	0.14
Female	14/26	53.8	63/260	24.2	4.3	1.8 to 10.4	
Age category (at index date)							
18 to 39 years	49/80	61.3	140/788	17.8	10.8	6.0 to 19.3	
40 to 49 years	60/97	61.9	168/946	17.8	8.1	5.1 to 12.9	0.29
> 50 years	27/47	57.4	103/459	22.4	5.4	2.8 to 10.3	
Urbanisation level^b^							
High urban (>2,500 addresses per km2)	42/76	55.3	117/633	18.5	7.4	4.2 to 13.0	0.49
Low urban (<2,500 addresses per km2)	58/83	69.9	201/948	21.2	9.7	5.7 to 16.2	
Missing^c^	36/65	55.4	103/612	16.8			

^a^ *n* = number of cases or controls with HIV indicator conditions, *N* = total number of cases or controls, % = percentage with HIV indicator condition

^b^High urban: >2,500 addresses per km2; low urban: < 2,500 addresses/km2

^c^Subjects with missing urbanisation level were not included in the analysis

^d^additional effect of gender/age/urbanisation level on the likelihood for an HIV-diagnosis after presence of HIV indicator conditions

### HIV testing in persons diagnosed with STIs

In 32.1% (95% CI: 17.9 to 50.7) of persons diagnosed with syphilis (9/28 of the cases and controls) no HIV test was reported in the EMRs in the same or follow-up consultation(s) within a time window of three months; for gonorrhoea this was 44.7% (95% CI: 30.1 to 60.3), (17/38), for hepatitis B 61.5% (95% CI: 35.5 to 82.3), (8/13) and for chlamydia 54.2% (95% CI: 40.3 to 67.4), (26/48).

## Discussion

Since 2012, European guidelines recommend IC-guided testing in primary care. This new strategy is also mentioned in the updated Dutch national STI guideline for GPs which was published at the end of 2013. Our findings strengthen the evidence for implementation of this new strategy as we showed that ICs were more commonly diagnosed in HIV-positive persons than in matched controls, in a period up to five years prior to HIV diagnosis. HIV testing after detection of other STIs has already been recommended for years. However, our results showed that an HIV test is not always performed according to the medical files. Our data showed no indication that the IC-guided testing strategy would have a different effect in sex- or age-based subgroups or in high-prevalence areas.

### Discussion of findings

A Spanish cross-sectional study showed that offering HIV testing to patients diagnosed with an IC could be cost-effective in primary care since the prevalence of HIV in these patients was higher than 0.1% for most of the suggested ICs [28]. Also, this Spanish study showed that ICs were frequently diagnosed by the GP, however, HIV testing occurred in only 18.6% of the patients with ICs. A Dutch case-control pilot study showed that more than half of all cases (58.5%) in a high-prevalence area had one or more ICs in the five years prior to HIV diagnosis [21]. A UK general practice case-control study found that 25.8% of HIV cases had presented with an ICs one year prior to the HIV diagnosis date [20]. In our study, 44.2% of cases had one or more ICs one year prior to the HIV diagnosis date. Also, a high number of cases were from highly urbanised areas, which are known to have a higher percentage of migrants [29]. The large numbers of local residents who originate from countries where HIV is endemic, and who are known to present to care late, may well account for the higher percentage of ICs [2, 21]. This provider-initiated strategy could be helpful in finding newly diagnosed HIV patients as early as possible.

In a qualitative study, Dutch GPs suggested that implementing the IC-guided testing might be more beneficial in a higher HIV-prevalence area than in areas with a lower HIV-prevalence [30]. In our study, no significant differences were seen in the strength of the association between HIV diagnosis and ICs when comparing genders, age groups or urbanisation levels. Our study suggests that IC-guided testing is not restricted to high-prevalence areas or subgroups.

Dutch national GP guidelines recommend that persons in high-risk groups should be tested for all five major STIs, namely chlamydia, gonorrhoea, hepatitis B, syphilis and HIV [17]. In our study, 36.6% of the cases had one or more STI(s) up to five years prior to diagnosis compared to 3.0% of the controls. The European guidelines recommend HIV testing for all STIs with an undiagnosed HIV prevalence of > 0.1% [18]. This implies that all patients in the general population with an unspecified STI diagnosis should be tested for HIV. In the Netherlands, persons who are diagnosed with an STI in primary care should be evaluated in terms of patient’s risk-assessment and symptoms to determine whether they should be further tested for STIs [11, 17]. Chlamydia is highly prevalent among Dutch patients who are in groups that are presumed to have a low risk of HIV. There is still debate about the need to test for HIV in primary care if chlamydia is found in a person not classified as being in a high-risk group [9]. Also, in our study the ORs for gonorrhoea and syphilis were higher than for chlamydia.

Acute HIV infection is a syndrome that can present as a mononucleosis-like illness, and therefore its detection can lead to the detection of HIV as early as possible. Mononucleosis-like illness includes such symptoms as fever, malaise, skin rash and generalised lymphadenopathy [31, 32]. Mononucleosis-like illness was reported in 1.3% of HIV cases in the five years prior to diagnosis compared to 0.3% of the controls. The low percentage found in the case group could be explained by the fact that a mononucleosis-like illness is not always documented in the EMRs. Also, GPs are not always aware of this acute syndrome [31, 32]. To increase the detection of acute HIV infection, GPs could be better informed about mononucleosis-like illness and the possible relationship with acute HIV infection.

### Strengths and limitations

This study was carried out in a GP network covering a representative sample of the Dutch population. Cases and ICs were searched for in the EMRs with the help of open source information extraction algorithms, which are known to significantly improve case detection when combined with codes [33].

The controls were matched with the cases to limit confounding. The selection of 10 controls per case ensured more statistical power to detect differences in rare events of ICs [26].

Reporting bias was minimised because the EMRs were based on observations that were routinely recorded before the HIV diagnosis was made. Therefore, there is no reason for differences between cases and controls in the accuracy of the information collected.

During data extraction we searched for ICs defined as ‘symptoms’ (e.g. fever) in the evaluation line of the consultation form (considered to be the final conclusion of the GP), to avoid the risk of overestimating this IC (e.g. fever often mentioned as complaint). As a result, an underestimation is likely and associations between symptoms and HIV diagnosis may be even stronger.

Not all ICs are likely to have been recorded in the EMRs [34]. STIs may have been diagnosed elsewhere. In the Netherlands, STI care is provided by GPs and STI clinics [7, 17]. Results from STI clinics are not always sent to the GP, which could cause an underestimation of the number of ICs [11]. For the same reason, the proportion not tested for HIV after an STI diagnosis may have been overestimated, since HIV tests may be performed at STI clinics. For example, we found that for 32% of persons diagnosed with syphilis, no HIV test was reported in the same or follow-up consultation(s).

If a person had multiple consultations for an IC, we used only the most recent one before the index date that was recorded by the GP. A limitation is that the information of repeated episodes was disregarded; a patient could have been diagnosed multiple times with the same IC prior to diagnosis, for example, multiple chlamydia diagnoses. We chose for this method because it was difficult to determine from the records whether a condition was diagnosed twice or if the same condition reappeared. This may have led to a slight underestimation of the associations between IC conditions and HIV diagnosis; however, we still found quite strong and significant associations.

Potential confounders or effect modifiers, such as ethnicity, sexual preference and socioeconomic status, could not be included in the analyses, because the standard information recorded in patients’ medical files does not include these characteristics [21]. In other European countries, too, sexual risk behaviour and sexual orientation are not included in EMRs in primary care [15, 16]. However, the strength of IC-guided testing is that it does not depend on patient characteristics: HIV tests are not offered specificly to groups labelled as being at risk and thereby avoids potentially complex conversations about sexual risk behaviour and/or ethnicity [15, 16, 35]. Therefore, ICs could help GPs to bypass some of the barriers presented by these conversations and thus help to normalise the use of the HIV test. [30]

## Conclusion

Our study showed that there are opportunities for IC-guided testing in primary care in the Netherlands. Unlike risk-based testing, this provider-initiated strategy is also applicable for all patients.

The majority of the ICs in the European guideline are not often seen by GPs, although all ICs are listed as relevant for HIV testing in primary care [18]. We recommend to determine a specific list of ICs that are more commonly seen in primary care and thus help GPs to implement IC-guided testing.

The context of pre-test information and how the HIV test is offered when an IC is diagnosed is important if patients are to agree to be tested [36, 37]. GPs should be educated about the context of pre-test information and how to offer an HIV test during IC-guided testing, for example, by using an opt-out approach (‘You will be tested unless you decline’). Many GPs use educational tools to keep up to date and these are often the first step in an implementation or change process [38]. We recommend that IC-guided testing should be better integrated in GPs’ future guidelines and that education strategies be developed to facilitate its implementation in daily practice.

## Additional file

Additional file 1: The proportion of persons classified by number of STIs and HIV indicator conditions in the period of one year or five years prior to the index date among HIV cases compared to matched controls. (DOC 54 kb)

## Abbreviations

CI: Confidence interval
EMR: Electronic patient medical record
GP: General practitioner
IC: HIV indicator condition
ICPC: International Classification of Primary Care
IPCI: Integrated Primary Care Information
OR: Odds ratio
STI: Sexually transmitted infection
UK: United Kingdom
UNAIDS: The Joint United Nations Programme on HIV and AIDS
