# Supplementary material for: The need to scale up HIV indicator condition-guided testing for early case-finding: a case-control study in primary care
Source: BMC Fam Pract. 2016 Nov 17;17:161. doi: 10.1186/s12875-016-0556-2 (PMC5114759; doi:10.1186/s12875-016-0556-2)
Supplement: Additional file 1: — The proportion of persons classified by number of STIs and HIV indicator conditions in the period of one year or five years prior to the index date among HIV cases compared to matched controls. (DOC 54 kb) [file 12875_2016_556_MOESM1_ESM.doc]

**Supplementary file**

**The proportion of persons classified by number of STIs and HIV indicator conditions in the period of one year or five years prior to the index date among HIV cases compared to matched controls**

|  | **Cases** (N=224) | | **Controls** (N=2,193) | |
| --- | --- | --- | --- | --- |
|  | **N** | **%** | **N** | **%** |
| *Number of STIs per person in one year prior to index date* |  |  |  |  |
| None | 174 | 77.7 | 2,169 | 98.9 |
| One | 36 | 16.1 | 22 | 1.0 |
| Two | 13 | 5.8 | 2 | 0.1 |
| Three or more | 1 | 0 | 0 | 0 |
| *Number of STIs per person up to five years prior to index date* |  |  |  |  |
| None | 142 | 63.4 | 2,125 | 96.9 |
| One | 54 | 24.1 | 60 | 2.7 |
| Two | 24 | 10.7 | 7 | 0.3 |
| Three or more | 4 | 1.8 | 1 | 0 |
| *Number of HIV indicator conditions per person one year prior to index date* |  |  |  |  |
| None | 125 | 55.8 | 2,032 | 92.7 |
| One | 67 | 29.9 | 142 | 6.5 |
| Two | 25 | 11.2 | 16 | 0.7 |
| Three or more | 7 | 3.1 | 3 | 0.1 |
| *Number of HIV indicator conditions per person up to five years prior to index date* |  |  |  |  |
| None | 88 | 39.3 | 1,782 | 81.3 |
| One | 71 | 31.7 | 340 | 15.5 |
| Two | 42 | 18.8 | 55 | 2.5 |
| Three or more | 23 | 10.2 | 16 | 0.7 |
